# Pseudospark-sourced beam and its application in high-power millimeter-wave generation

**DOI:** 10.1038/s41598-021-98564-x

**Published:** 2021-09-24

**Authors:** Liang Zhang, Huabi Yin, Wenlong He, Xiaodong Chen, Jin Zhang, Adrian Cross

**Affiliations:** 1 Department of Physics, SUPA, University of Strathclyde, Glasgow, G4 0NG UK; 2 College of Electronic Science and Technology, Shenzhen University, Shenzhen, 518060 China; 3 School of Electronic Engineering and Computer Science, Queen Marry University of London, London, E1 4NS UK

**Keywords:** Plasma physics, Electronic and spintronic devices

## Abstract

A pseudospark (PS) discharge can generate an electron beam with a high current density. The electron beam can be self-focused by an ion channel and transported over a long distance without the need for an external magnetic field. Such features make it attractive to drive millimeter-wave/THz interaction circuits for the generation of high-power radiation from a compact device. This paper presents the experimental results on the generation and transportation of the PS-sourced beam with different cross-sections, as well as the differences of the PS-sourced beam with and without post acceleration. Its application in millimeter-wave/THz sources was demonstrated by the operation of extended interaction oscillators (EIOs) at different frequencies is presented.

## Introduction

The pseudospark (PS) discharge was first studied by J. Christiansen in 1978^1^. It is a form of low-pressure gas discharge in the range of 50 mTorr to 500 mTorr, and the discharge process is characterized by a rapid breakdown phase and the generation of an electron beam with a high current density up to $10^4 \mathrm{A}/\mathrm{cm}^{2}$. The discharge current can reach tens of kiloamperes with a rise time of a few nanoseconds. Therefore, the PS discharge can be used as a high current switch in high-power pulsed circuits. The electron beam extracted from the PS discharge chamber can be used in applications such as material treatment, lithography, X-ray generation and plasma jet^2–6^.

The unique features of the PS-sourced beam are attractive in the generation of high-power millimeter-wave and terahertz (THz) radiation^7–9^. The current lack of high-power and high-quality millimeter/THz sources is one of the major barriers that limit their applications. In the literature, the most promising approach to generate high-power millimeter/THz radiation is through the resonance of a free electron beam with a high-frequency interaction circuit. The biggest challenge is the small dimensions of the beam-wave interaction circuit at the high operating frequency. The typical slow-wave interaction circuit for single-mode excitation at 100 GHz has a radius of $\sim 1\ \hbox {mm}$. The beam tunnel to cut off the wave has a smaller diameter therefore it limits the cross-section of the electron beam. In millimeter-wave vacuum electronic devices, thermionic cathodes are normally used. The small beam cross-section and the relatively low current density limit the overall current and lead to low output power.

There are ways to increase the current density and power capability. For example, the photocathode^10^ can produce a current density a hundred times that of the thermionic cathode, however it suffers from a short lifetime and requires a high-power laser as well as an ultra-high vacuum environment. The gyrotron devices based on the fast-wave cyclotron maser instability^11^ can have a much larger dimension of the interaction circuit and therefore a much large beam current, however they require a strong magnetic field $\sim f(\mathrm{GHz})/28$ in Tesla for the beam-wave interaction when operating at the fundamental electron cyclotron frequency. For millimeter and THz gyro devices, a superconducting magnet is normally required which is not suitable for a compact and low-cost solution.

The PS discharge operates at room temperature. The structure is simple and has an excellent lifetime. The PS-sourced beam has a current density $\sim 100$ times larger than the thermionic cathode. When it is transported in a low-pressure gas, its pulse front will ionize the background gas to generate plasma with a density comparable to the beam density. The ionized electrons have larger velocities and are expelled by the beam electrons to leave an ion channel. For optimum discharge conditions, the electron beam can be focused by the ion channel and transported a long distance. While in other vacuum electronic devices, an external guiding magnetic field either generated from a periodic permanent magnet or a solenoid is needed to compensate the space charge force of the electron beam and it is difficult to focus the beam through the device. The self-focus feature of the PS-sourced beam not only eliminates the bulky magnet system and also greatly simplifies the device to achieve a compact design but also relieves the tight alignment requirements of the magnetic field and the electron beam at high operating frequencies.

Various experiments were carried out to demonstrate the application of the PS-sourced beam in millimeter-wave sources at different frequencies^12–18^. The electron beam generated at different PS discharge stages, and the radiation powers from extended interaction oscillators (EIOs) using different beam cross-sections were investigated. The results are summarized in this paper.

## EIO interaction circuit

### Design of the EIO interaction circuits

Different millimeter-wave interaction circuits were investigated, including the Cherenkov maser^9^, the backward wave oscillator^12^, and the extended interaction oscillator (EIO)^19^. Among these interaction circuits, the EIO has the advantages of high gain per unit length, high electronic efficiency, high radiation power, and a compact structure. Therefore it attracts significant interest as the millimeter-wave source. At present, the commercially available EIOs by Communications & Power Industries (CPI) company at 94 GHz can produce an output power of 80 W at continuous-wave (CW) mode and 1.5 kW in pulsed power mode. At 220 GHz and 263 GHz, the peak powers are 100 W and 70 W in pulsed power mode, respectively^20–23^. As the frequency increases, their output power drops dramatically. To further improve the output power, EIOs driven by sheet electron beams were developed^24,25^. A W-band sheet-beam EIO demonstrated by Naval Research Laboratory achieved a peak output power of 7.5 kW^24^. To reduce the complexity of the magnet system in the sheet beam devices, a multiple pencil beam configuration was also proposed^26^. Other EIO research in recent years include increasing the bandwidth of the EIO using mode switching by adjusting the beam voltage, and higher-order mode operation to increase the dimensions of the interaction circuit^27,28^.

**Figure 1:**
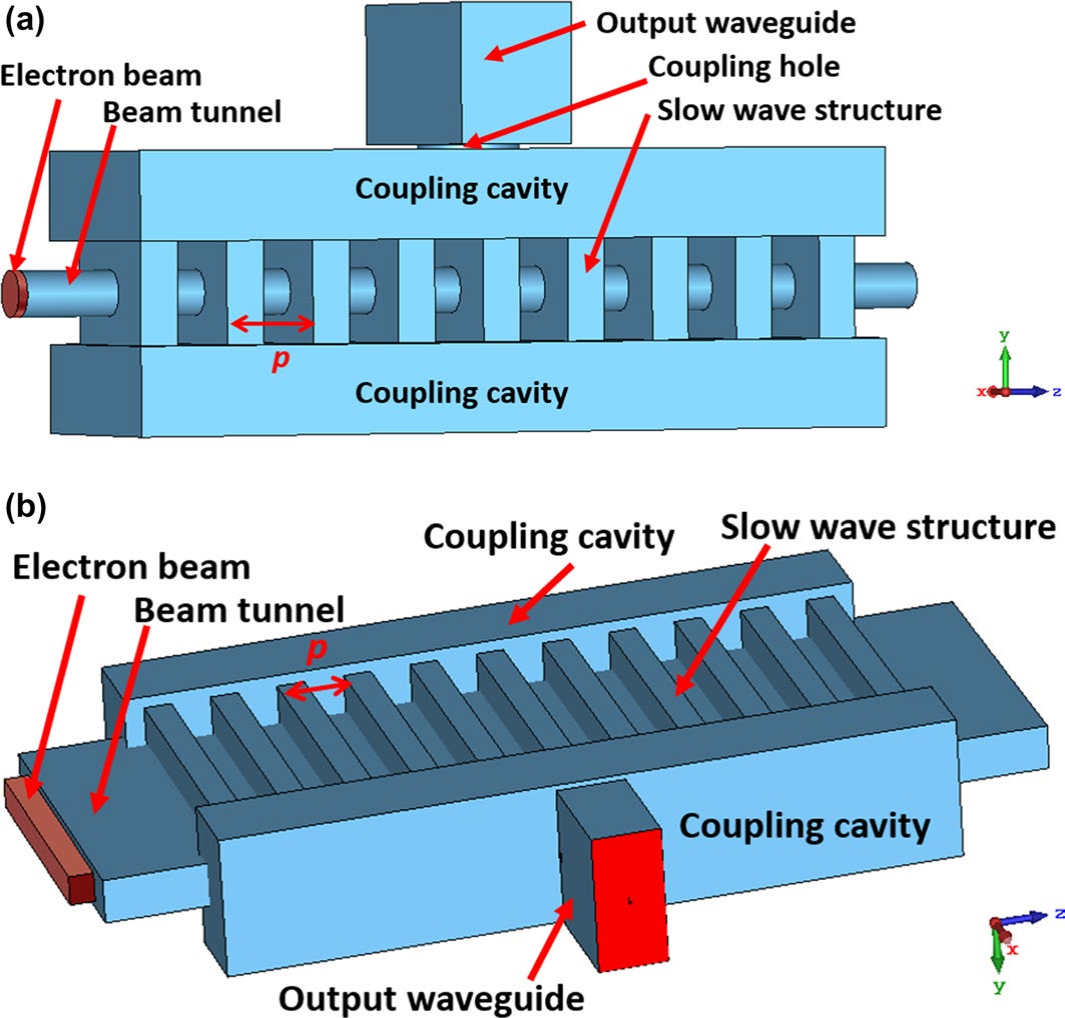
The schematic drawings of a pencil-beam EIO (**a**) and a sheet-beam EIO (**b**). Both drawings show the vacuum enclosures of the EIO structures.

Both pencil beam and sheet electron EIOs, as shown in Fig. 1, were designed. For the EIOs with thermionic cathodes, the differences between the pencil beam and sheet beam are significant, from the cathode shape to the guiding magnetic field. The sheet electron beam has less space charge force and can achieve a larger total current. It has the advantage to achieve larger radiation power in the millimeter-wave and THz frequency range. However it has a more complicated magnet design and a tighter tolerance requirement compared with the pencil beam. It is much easier to generate and transport the PS-sourced beam due to the ion-channel self-focusing.

From theory, there are no significant differences in the design of EIO structures for both types of electron beams. The resonance condition between the electron beam and the interaction circuit can be expressed as

1 $$\begin{aligned} 2f/v_e =m/p \end{aligned}$$

where *f* is target operating frequency, $v_e$ is the velocity of the electron beam, and *p* is the period of the EIO structure. Normally $2\pi$ mode ($m=2$) is chosen because it has the largest shunt impedance (*R*/*Q*). After the period has been decided from the target operating frequency and the beam voltage, the electron’s velocity can be determined allowing the other dimensions to be optimized using the eigensolver in CST Microwave Studio. The aims were a large beam voltage separation of the competing modes ($2\pi -1$ mode and $2\pi +1$ mode), an eigenfrequency of f of the $2\pi$ mode, and the maximum shunt impedance.

The beam-wave interaction of the designed EIO was simulated by 3D particle-in-cell (PIC) software CST Particle Studio^29^. In the simulation, a constant magnetic field was used to provide an equivalent focusing force to the ion channel. The plasma background in the interaction region was considered with an effective dielectric constant.

2 $$\begin{aligned} \varepsilon _r=1- \omega _{pe}^2 / \omega ^2 \end{aligned}$$

where $\omega _{pe}$ and $\omega$ are the plasma oscillation frequency and wave frequency, respectively. The frequencies and powers of the radiation at different beam conditions and the tolerance of key dimensions were also studied.

### Manufacture of the EIO interaction circuits

**Figure 2:**
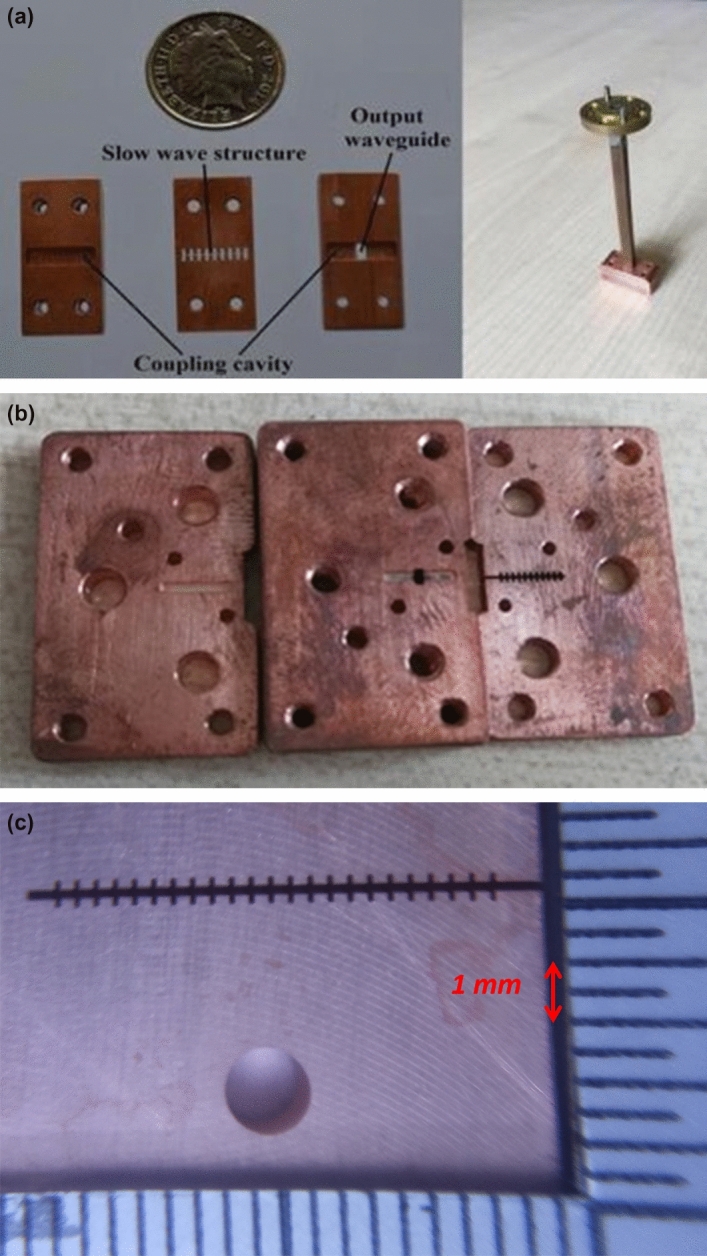
The machined parts of the EIO structure. (**a**) W-band EIO and its assembly, (**b**) The G-band EIO parts before assembling, (**c**) The wire cut 350 GHz slow-wave structure in the major EIO parts.

The designed EIO was split into three parts, the slow-wave structure, the coupling cavity, and the output waveguide. The slow-wave structure, which is a planar structure, was machined by a wire cutting technique using $\Phi 30 \mu \mathrm{m}$ tungsten wire. The other two parts were machined by a CNC machine and spark erosion for the coupling slots. The machined parts were dipped in an acid etch to remove the oxide layer and to achieve a better surface finish. Figure 2 shows the photos of the different EIOs (W-, G- and Y-bands) at different manufacturing stages. Figure 2a is the manufactured parts and the assembled structure of a W-band pencil-beam EIO operating at 94 GHz. Figure 2b is the machined parts of the G-band (200 GHz) sheet-beam EIO before the bright dip acid etch. Figure 2(c) is the slow-wave structure of a 350 GHz sheet-beam EIO after bright dipping.

## Experimental setup and diagnostics

### PS discharge experimental setup

**Figure 3:**
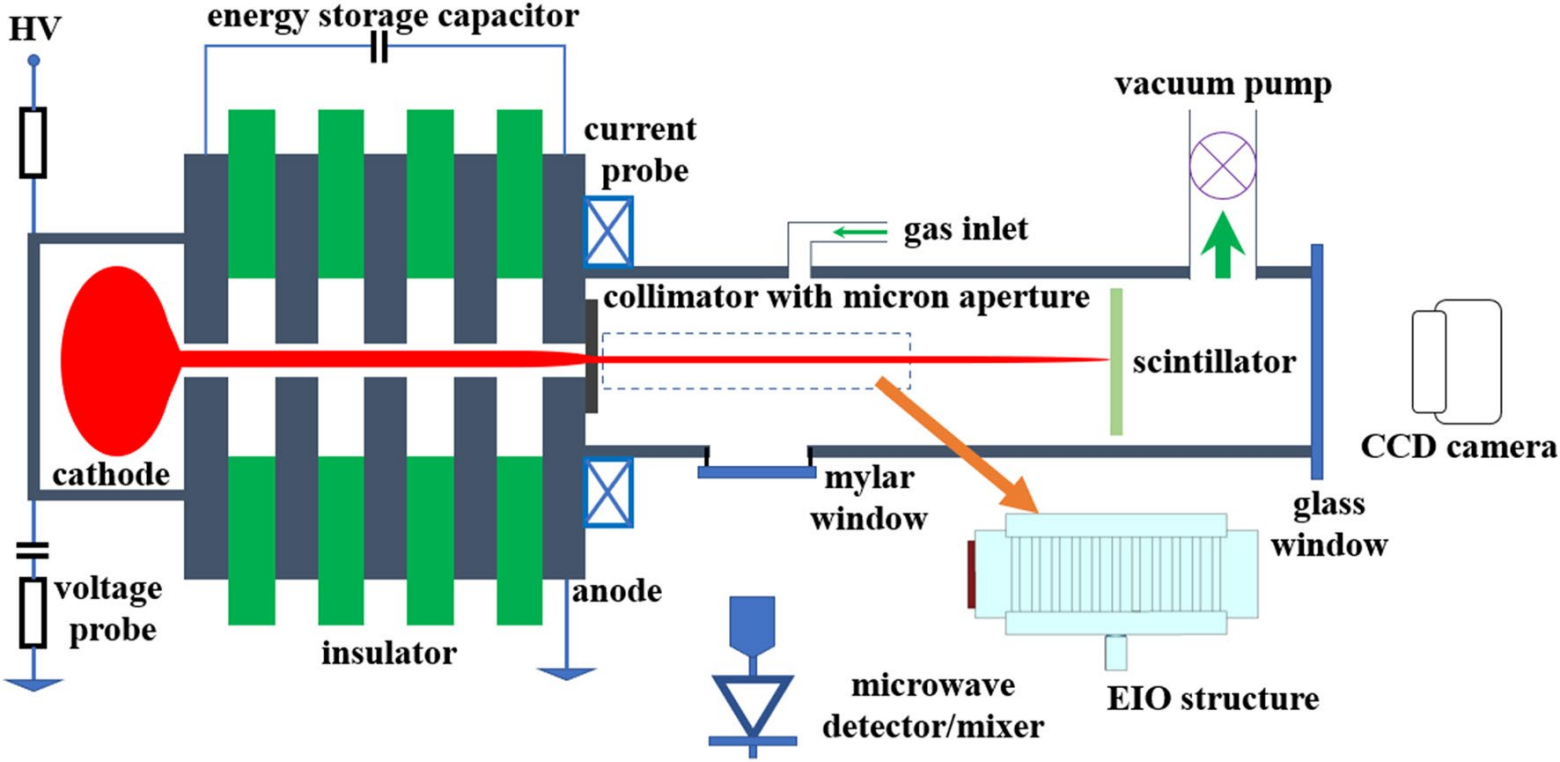
The schematic of the PS discharge experimental setup.

Figure 3 shows the experimental setup of the PS discharge^30^. A hollow cavity made of stainless steel with a centric hole was used as the cathode. Argon gas was used in the experiment. The gas pressure in the discharge cavity could be finely adjusted by the combination of the needle valve in the gas inlet and the vacuum valve beside the vacuum pump. The anode was a planar plate with a centric hole in which the generated electron beam was extracted. The cathode was charged to a negative high voltage, and the anode was grounded. Between them was a dielectric disc to insulate the high voltage. High-voltage capacitors of 470 pF were used to store the energy and to maintain the discharge voltage.

Based on the geometry of the insulators between the cathode and anode, the PS discharge has two configurations, single-gap and multi-gap configurations. Both of them have been studied through experiments on the breakdown process as well as the discharge properties. When operating at a low voltage (about a few kilovolts), a single dielectric disc is normally used. A high-energy electron beam can be generated from a multi-gap configuration, where multiple dielectric discs are placed in turn with copper discs. At a high operating voltage (larger than 20 kV), the multi-gap configuration can avoid the flashover in a long dielectric tube, and also helps to create a uniform electric field distribution to achieve a more stable discharge process at a high repetition rate operating mode. An electron beam with an energy of 200 keV was generated by a 10-gap configuration in 1995^31^. In simulation studies, the major focus in the previous research was on the single-gap PS discharge configuration. In recent years, the multi-gap PS discharge mechanism was studied through PIC simulations on the uniform electrode apertures^32^. The discharge current as the function of gap pressure was also simulated under irregular electrode apertures, as well as rectangular apertures to generate a sheet electron beam directly^16,33,34^. Based on these studies, it is feasible to generate electron beams with desired cross-sections for different applications.

The PS discharge can operate either at the self-breakdown or triggered-based mode. Both operating modes have been studied in experiments, with the triggered-base mode investigated in simulations^35–38^. At self-breakdown mode, the voltage between the cathode and anode, or the gas pressure inside the discharge cavity will be adjusted slowly to reach the breakdown condition. The PS discharge process ignites randomly by the avalanche ionization of the background gas by the free electrons, whose density is around $10^9$ to $10^{10}\ \hbox {m}^{-3}$ in the gas due to ultraviolet and cosmic radiation. The self-breakdown is useful to characterize the discharge conditions of the studied PS structure, for example, to determine the voltage-pressure curve. The discharge process is fully developed therefore typical PS discharge voltage and current waveforms can be observed. However, the jitter of the breakdown in the self-breakdown mode is relatively large and the time delay can be long. Small jitter can be achieved by operating the PS discharge in the triggered-based mode. The seed electrons to initiate the discharge process can either be produced electrically by applying a high-voltage pulse to a sharp needle or optically by a laser signal. The previous simulations and experiments showed that the number of seed electrons needs to be of the order of $10^{10}$ to sustain the avalanche breakdown. The triggered-based PS discharge is preferred in applications that require repetition rate operation.

The beam energy of the EIOs presented in this paper was around 30 kV. A 4-gap configuration was used in the experiment and the PS discharge operated at self-breakdown mode. After the electron beam was extracted from the anode aperture, a collimator with a rectangular slot or a circular hole in the center was used to shape the electron beam into a pencil beam or a sheet beam, depending on what kind of interaction circuit was being used. The waveform of the discharge voltage was measured by a resistor-type voltage divider, and a Rogoswki coil with nanosecond response time to measure the discharge current. A moveable Faraday cup was used to measure the beam currents at different distances to the collimator. The beam profile was screened by a scintillator and the image was captured by a CCD camera placed at a fixed distance to the scintillator. The beam cross-section and the beam current can only be measured separately as they will stop the electron beam.

After characterizing the PS-sourced beam, the interaction circuit was connected with the collimator to generate millimeter-wave radiation. The output power was extracted out by a standard pyramid horn and the pulse shape and power level were measured by in-band crystal detectors from Farran. The W-band crystal detector was calibrated using a broadband Quinstar solid-state source which can generate 1.5 W output power between 90 to 97 GHz. Firstly, the output powers of the solid-state source at different frequencies were measured using a tunable attenuator and a W-band power meter. Then the power meter was swapped with the crystal detector. Its response to different input power levels at different frequencies were recorded. In the EIO experiments, the radiation power was transmitted and collected by a pair of standard pyramid horns separated by a distance of 20 cm. Additional calibration was done by inserting the pyramid horns between the solid-state source and the crystal detector. The detector responses at the different distances between the horns were also recorded. The EIO radiation power was calculated from the two sets of calibration data. A G-band source with a large output power was unavailable. Therefore the G-band crystal detector was calibrated using a vector network analyzer (VNA) with a maximum output power of 10 dBm operating in DC output mode. The same calibration process was used as described for the W-band experiment. The calibration of the G-band detector was not as accurate as the W-band calibration due to the small amplitude of the detector signal as a consequence of the limited by the output power of the VNA. The maximum measurement error in G-band was about 20%.

The radiation frequency at W-band was measured by cut-off filters. The cut-off filters were a set of straight circular waveguides machined by wire cutting technology. The waveguide diameters were in the range of 1.76 mm to 1.94 mm, corresponding to the cut-off frequencies from 99.8 GHz to 90.6 GHz which was measured using a W-band VNA. In the experiments, the cut-off filter was connected between the pyramid horn and the crystal detector. The radiation generated from the EIO could only be detected by the detector if its frequency was higher than the cut-off frequency of the filter. The accuracy of the frequency measurement was determined to be accuracy of the waveguide diameter, which was 0.02 mm corresponding to 1.0 GHz difference at the respective cut-off frequencies.

## Measurement results

### Discharge voltage and beam current

**Figure 4:**
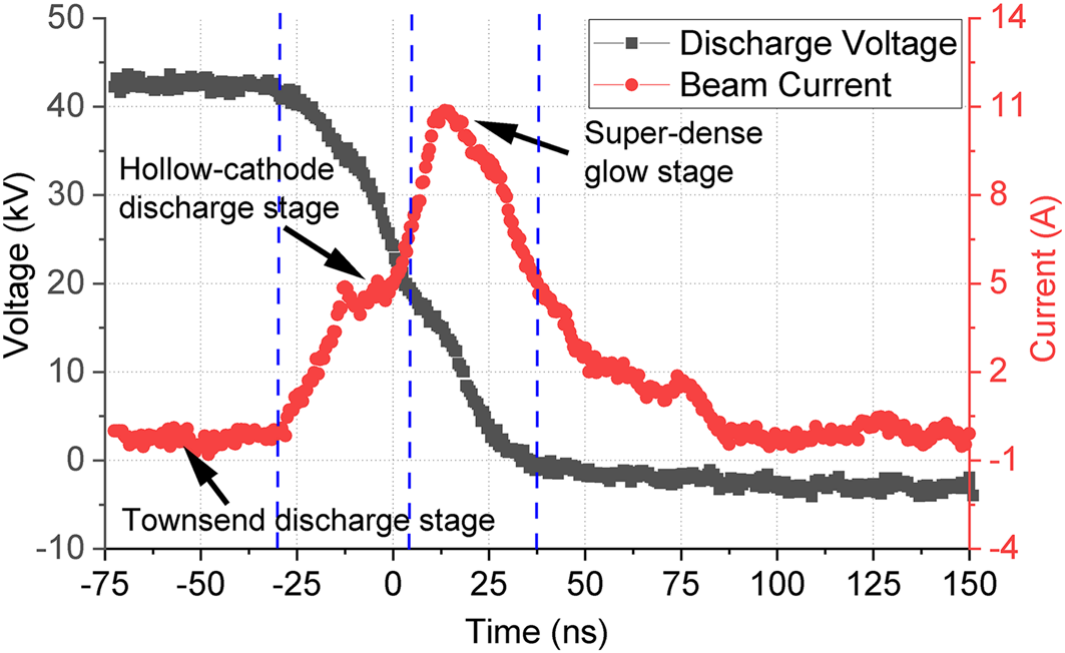
The correlated waveforms of the discharge voltage and the electron beam.

In the experiment shown in Fig. 3, the PS discharge used a 4-gap configuration and it operated at self-breakdown mode. The gas pressure in the discharge cavity was fixed and the charging voltage at the cathode was increased slowly until the breakdown occurred. The PS discharge process can be divided into three phases, including the Townsend discharge phase, the hollow cathode discharge phase, and the super-dense glow discharge phase. Figure 4 shows the typical waveforms of the discharge voltage and the correlated beam current. The discharge stages can also be identified from the beam current, as labeled in Fig. 4.

When the discharge voltage was 42 kV, the beam voltage at the hollow-cathode discharge stage was in the range of 35 - 20 kV which matched with the operating beam energy of the EIO, and the beam current was about 4 A. The maximum beam current in the super-dense glow stage was 11 A, which was about 3 times larger than the hollow-cathode discharge stage. However, its beam energy was low and did not satisfy the beam-wave resonance condition as stated in Eq. (1).

The dynamic of the discharge process was simulated using 2D PIC-MCC code XOOPIC^39^. It provided more details on the beam quality. The beam energy spread at the hollow-cathode discharge stage was about 20%, while the energy spread at the super-dense glow stage was about 40%. Further PIC simulations of the EIO beam-wave interaction showed the resonance would not build if the beam energy spread is greater than 35%. The beam energy spread in the condense discharge phase was too large and was not suitable for beam-wave interaction. Only the electron beam generated in the hollow-cathode discharge phase could be utilized.

**Figure 5:**
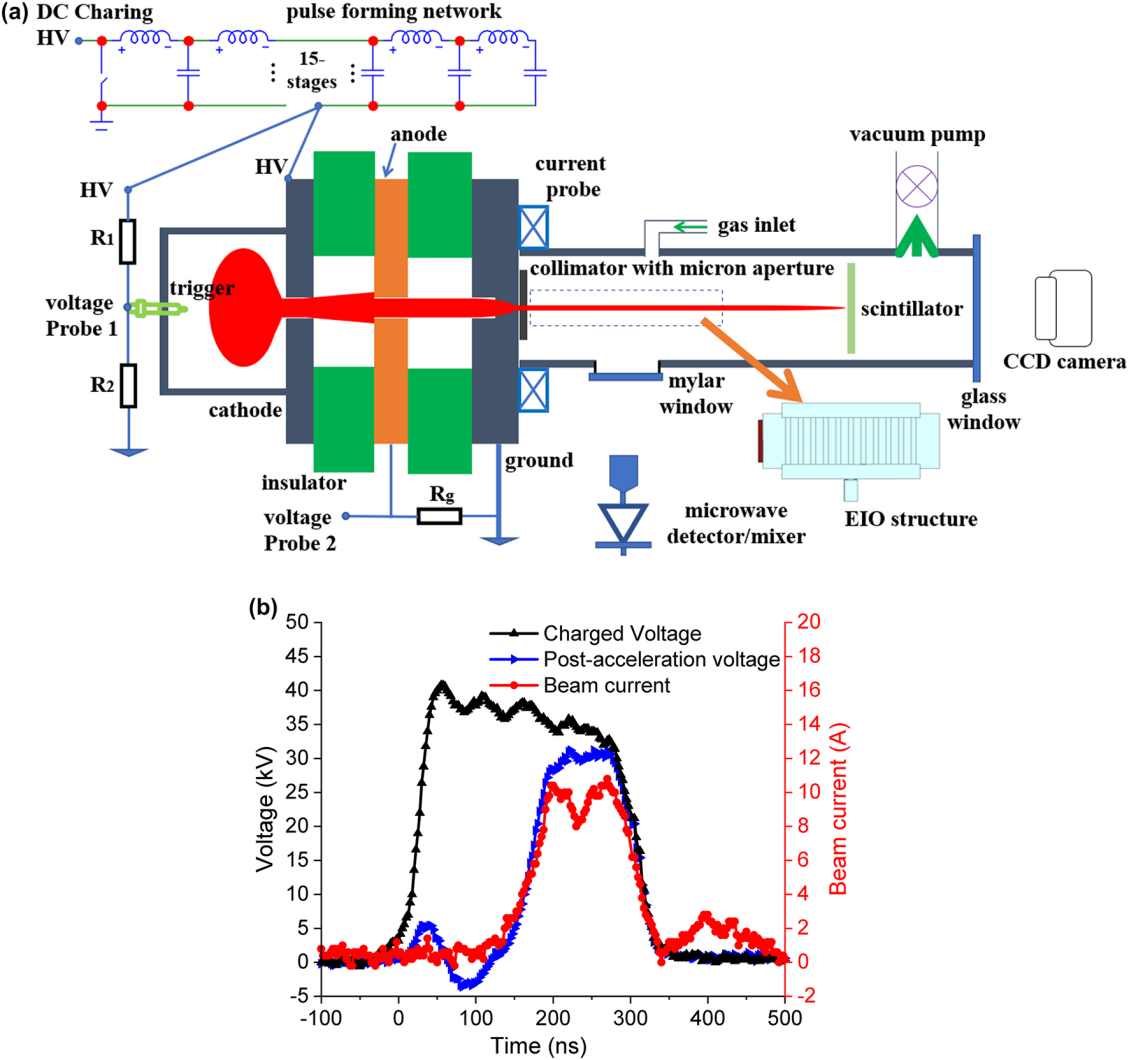
(**a**) The schematic drawing of the hollow-cathode discharge experimental setup with post-acceleration, and (**b**) The waveforms of the discharge voltage and acceleration voltage pulses.

The low energy and large spread of the electron beam in the super-dense glow phase can be improved by post accelerating^40,41^ to higher energy. The beam will have a larger current density therefore increasing the output power. However, the anode should be at floating potential and an additional power supply is required. The acceleration pulse also needs to match with the breakdown voltage, which requires a dedicated circuit design. It is challenging to achieve high stability in generating the electron beam at a high repetition rate. A novel hollow-cathode discharge setup with a pulse forming network (PFN) was proposed to simplify the design^41^. As shown in Fig. 5(a), a trigger voltage pulse was generated from the voltage divider composed by $R_1$ and $R_2$ to provide the seed electrons and to ignite the discharge process. Different from the operating conditions in Fig. 3, in this experiment a low-voltage high-current trigger was used to achieve a fast super-dense glow discharge phase. The low-energy electron beam extracted from the anode aperture was accelerated between the anode and the ground plate. Since the voltage between the cathode and anode was low, a single-gap configuration was used. The gap between the cathode and anode broke down in a short time. Because the anode had a floated potential, it was driven to the same potential as the cathode after the breakdown. As the beam energy was low, the cathode was still able to maintain a high voltage. Therefore the electron beam could be accelerated to high energy. Typical discharge voltage, post-acceleration voltage, and beam current are shown in Fig. 5(b). With post-acceleration, the beam current for the beam-wave interaction was doubled compared with the case without post-acceleration. The electron beam pulse also had a longer duration.

### Scintillator screening and beam transportation results

**Figure 6:**
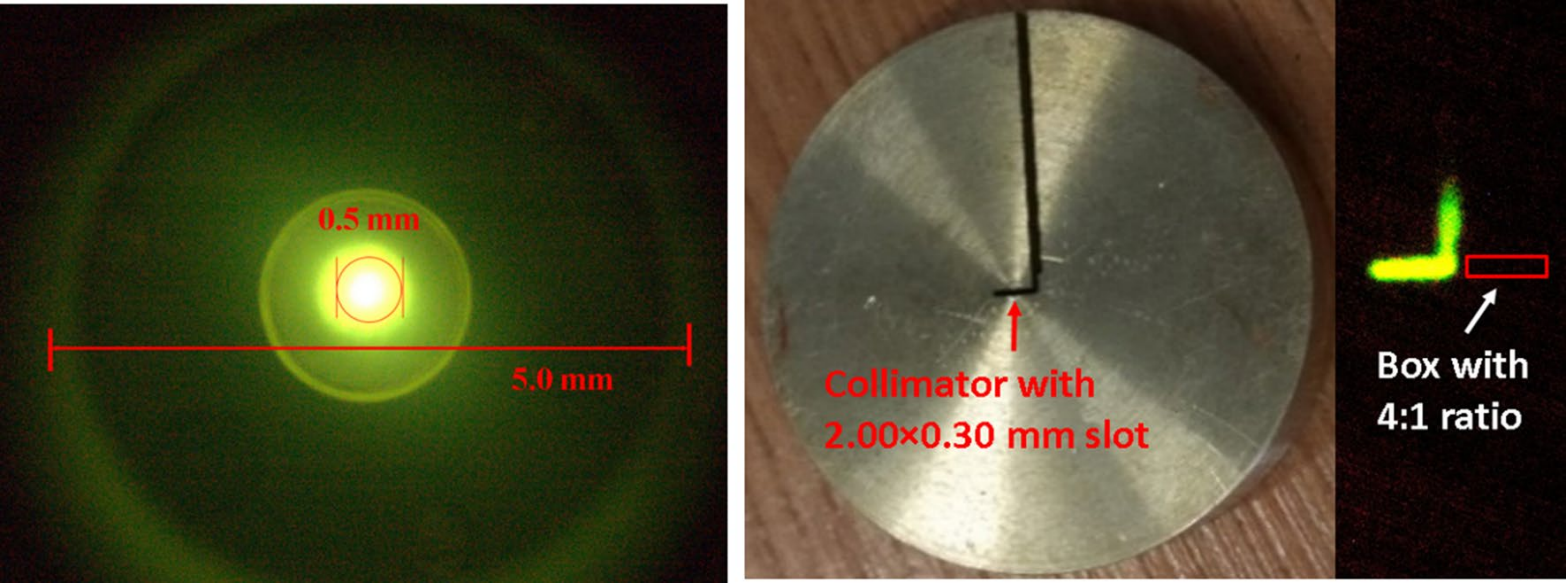
Images of pencil beam (left) and sheet electron beam (right).

Collimators with a centric hole and a rectangular slot were used to generate the pencil electron beam and sheet electron beam. Their cross-sections were measured by a scintillator and a CCD camera. Figure 6 (left) shows the image of a pencil beam. Post-processing results showed the electron beam current had a full-width half-height (FWHH) of 0.5 mm^42^. Figure 6 (right) shows the collimator with a rectangular slot of 2.00 * 0.30 mm. The correlated beam cross-section was obtained with post acceleration. The width-to-height ratio of the measured beam was about 4:1, which was smaller than the collimator slot ratio. It was caused by the transverse velocity and the space charge effect. A relatively low width-to-height ratio slot can therefore be used to help the formation of the ion channel since a high aspect ratio slot did not seem to help.

**Figure 7:**
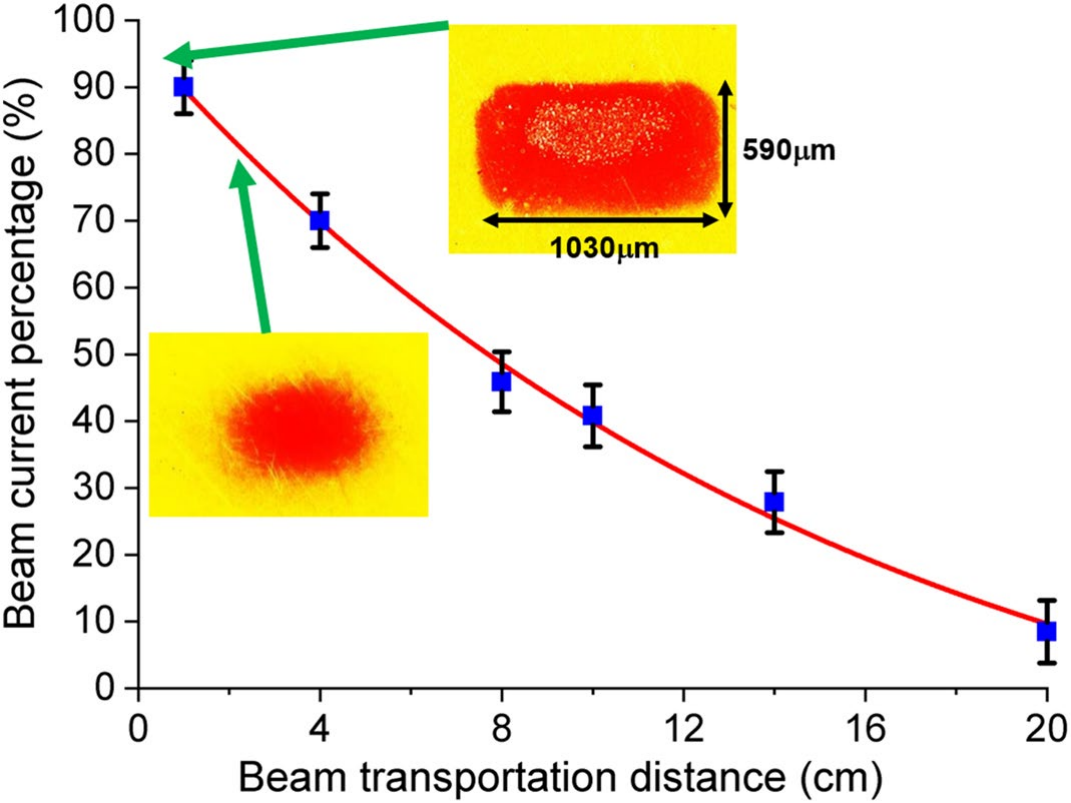
Beam transportation and the cross-sections of the sheet beam at different positions.

The transportation of the electron beam in a large drift tube was measured by a Faraday cup. Without the guiding magnetic field, 10% of the electron beam could still transport a distance of 20 cm from the anode, as shown in Fig. 7. The loss of beam current was mainly caused by the collisions between the electrons and the background gas. At a transportation distance of 40 mm, which was approximately the length of a millimeter-wave interaction circuit, the beam current percentage was about 70%. The preservation of the beam cross-section was also studied. It was more challenging to transport a sheet electron beam than a pencil beam as different focusing forces in both transverse directions were needed. Figure 7 also shows the images of a sheet electron beam at different positions. With a drift distance of 15 mm, the sheet electron beam still kept its shape however the distortion at the edges was also more pronounced.

The energy and velocity spreads of the electron beam during transportation were important parameters for the EIO interaction. However, they could no be obtained directly from the above measurements. The theoretical model showed the beam would over-focus if the current density was too low, or it would defocus if the current density was too high. The rest electrons in the ion channel could also cause betatron instability if its density was large^43^. All these phenomena would increase the beam velocity spread. Stable beam transportation could be achieved for suitable parameter ranges. In experiments, it required choosing the PS discharge conditions carefully such as gas pressure and charging voltage.

### EIO results

PIC simulations were used to simulate the output frequency and power level of the EIO interaction circuit using CST Particle Studio. In the simulations, the collisions between the electron beam and background gas were not considered, because CST Particle Studio does not have a Monte-Carlo collision model integrated into the PIC solver. Also the collisions and ionization process will significantly increase the simulation time due to the rapid increase of the particle number. Instead, the EIO driven by different beam currents that takes into account the beam lost due to collisions were simulated. When operating at higher frequencies, the dimensions of the EIO structure became smaller. The dynamic of the PS-sourced beam would have a more significant impact on EIO’s performance. For example, the beam tunnel size was 1.3 * 0.18 mm in the 350 GHz sheet-beam EIO. The formation of the ion channel and the stability of the beam transportation in small beam tunnels are still open questions. It is difficult to get an accurate prediction of the output power from the PIC simulation. However a few factors could be considered to get a reasonable prediction of the EIO performance. In the PIC simulation, the 0.35 THz sheet-beam EIO had an output power of 4.5 kW when driven by an ideal beam and with the conductivity of ideal Oxygen-free high conductivity (OFHC) copper. Varying the beam voltage from 32 to 36 kV would change the radiation frequency from 351.7 to 352.4 GHz without significant differences in the radiation power. The output power is proportional to current density. It was 1.8 kW considering the beam transportation and loss resulting in a current density value of $5 \times 10^7 \mathrm{A}/\mathrm{m}^{2}$. Taking into account the Ohmic loss and the manufacturing tolerance, the output power dropped to 305 W when 10% of the OHFC conductivity was used for the interaction circuit. The beam velocity spread would further reduce the output power. At a 20% energy spread, the EIO had 50 W output power in the worst scenario. It was found that the EIO would not work when the energy spread reached 35%.

**Figure 8:**
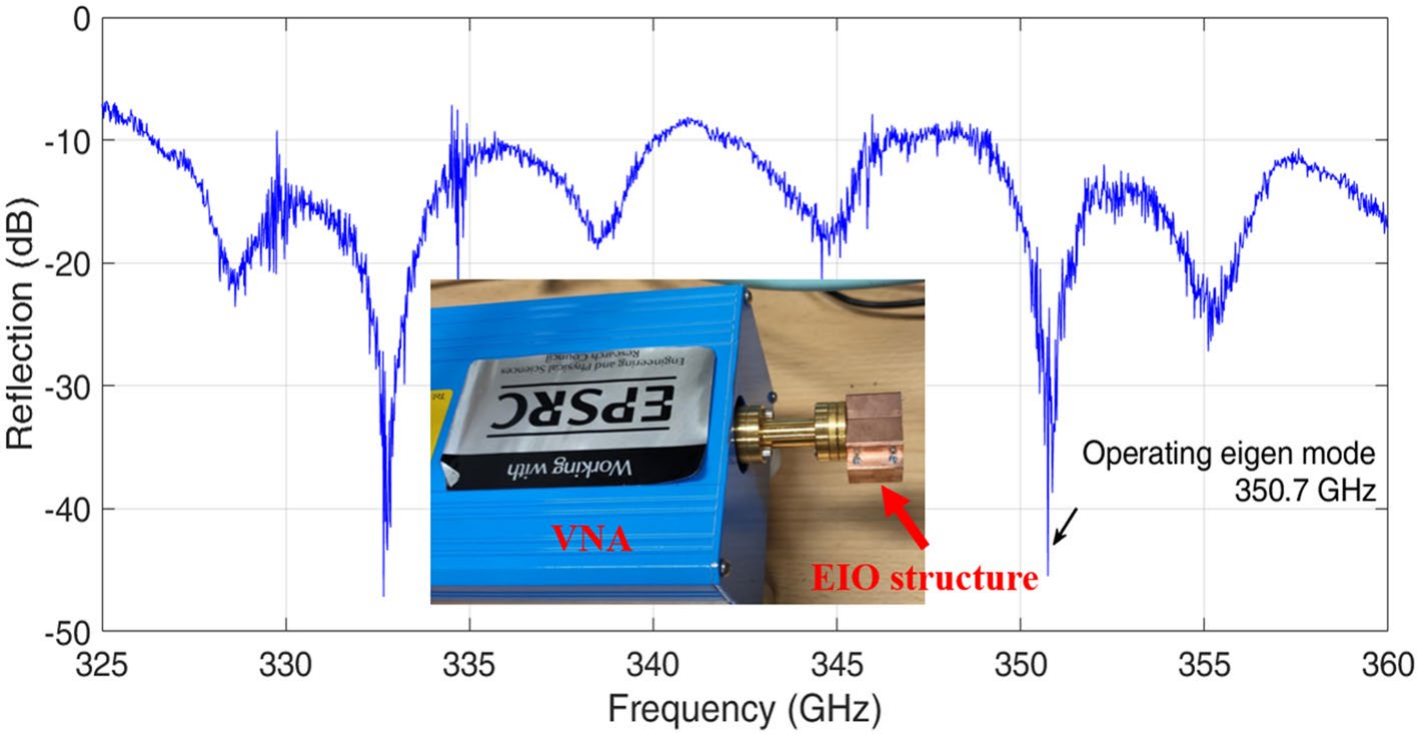
VNA measurement of the 350 GHz EIO.

The eigenfrequency of the EIO was measured by a vector network analyzer (VNA). The reflection coefficient of the 350 GHz EIO is shown in Fig. 8. The operating mode had a resonance frequency of 350.7 GHz, which is very close to the design value. The quality factor $Q_0$ in the measurement was 215, corresponding to a conductivity of $0.6 \times 10^7 \mathrm{S}/\mathrm{m}$ (10.3% of the OHFC conductivity). It matched with the prediction in the PIC simulations.

**Figure 9:**
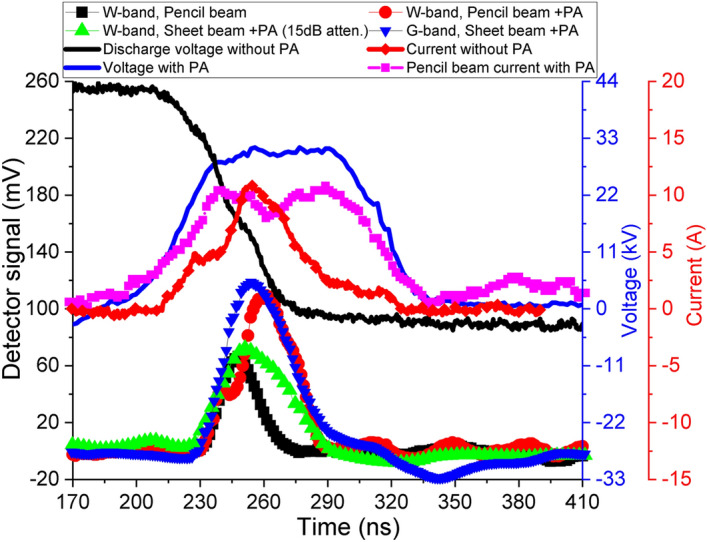
The measured millimeter wave signals in the experiments. A 15 dB attenuation was used between the detector and the receiving pyramid horn when measuring the W-band sheet-beam EIO with post acceleration.

A summary of the EIO’s results in the experiment is shown in Table 1. the measured voltages, currents and the millimeter wave pulses are shown in Fig. 9. It should be noted that it is the peak beam current measured by the Rogoswki coil right after the collimator. Since the beam tunnel of the EIO at a higher frequency is smaller than the aperture of the collimator, the beam current for beam wave interaction will be smaller than the values listed. The 94 GHz EIO with (i) pencil beam, (ii) pencil beam with post acceleration, (iii) sheet beam with post acceleration had an output power of 38 W, 200 W, and 1.2 kW, respectively. It was caused by the increase of the beam current of each beam type. At 200 GHz, the output power had a significant decrease. There were a couple of reasons. (1) The beam current was smaller due to the smaller beam tunnel. (2) The Ohmic loss was more significant at a higher frequency. (3) The beam quality became worse as it would be more difficult to form the ion channel in a smaller volume. These three factors were the major reason for the dramatic power reduction which will be more serious for the 350 GHz EIO. Therefore it is more important to investigate the formation of the ion channel and the beam propagation in the small beam tunnel before carrying out the measurements of the 350 GHz EIO.

**Table 1:** The experimental results of the developed EIOs.

Frequency (GHz)	Beam type	Beam voltage (kV)	Peak current (A)	Output power (W)
$>92$	Pencil beam	30.5	$\sim 5$	38
$>92$	Pencil beam with post acceleration	30.5	$\sim 10$	200
92-94	Sheet beam with post acceleration	32	$\sim 30$	1200
200	Sheet beam with post acceleration	34.5	$\sim 30$	10
350	Sheet beam without post acceleration	29.5	$\sim 30$	Not yet measured

## Discussion and conclusions

Compared with commercially available EIOs with a thermionic cathode electron guns by CPI, the EIOs driven by PS-sourced beam have comparable output power levels. However they eliminate the challenges in the beam optics design and the precise vacuum brazing to achieve ultra-high vacuum for the operation of the thermionic cathode of the EIO, due to the unique features of high current density and ion-channel self-focusing. It has demonstrated great potential in high-power millimeter-wave generation and the advantage to make compact devices is clear. Useful results from the research presented in this paper are summarized as follows.

1. Electron beams with different cross-sections can be generated with different collimator shapes. The sheet beam has a larger overall current than the pencil beam. However its cross-section distorted faster in the ion channel than the pencil beam. A sheet-beam EIO is preferable at higher frequencies as the overall current is large and the interaction circuit is shorter.
2. Post-accelerating the PS-sourced beam allows utilizing the beam generated at the super-dense glow phase. With post-acceleration, an electron beam with a larger current and smaller energy spread can be achieved. A higher radiation power can therefore be produced. However, post-acceleration requires a more complicated power supply circuit. It was also found in the experiment that the discharge process was less stable with post acceleration.
3. It should be noted that the PS-sourced beam has the disadvantage of low energy efficiency. Although the radiation power is high, its pulse duration is shorter than 100 ns and hence the average power is low. Operating at a high repetition rate helps to increase the average power. It requires stable parameters and the PS discharge without post acceleration is a better option.
4. The EIOs operating at W-band (94 GHz) and G-band (200 GHz) had been successfully demonstrated. The 350 GHz EIO structure was measured using a VNA. Future experiments will be carried out to measure the radiation power at 350 GHz. In general, operating at higher frequencies is more challenging due to the smaller beam tunnel. It is important to investigate the formation of the ion channel in small beam tunnels and to investigate the parameter ranges that allow stable beam transportation. PIC simulation will be carried out to investigate the limit of the beam tunnel, as it defines the highest frequency that the EIO can operate.
5. The stability of the PS discharge is a key issue for the millimeter-wave/THz sources in practical applications. An upgraded experimental setup is being developed to achieve improved stability. The improvements include a better vacuum-sealed discharge chamber, ceramic insulators that have better voltage hold-off strength, electric insulation and less outgassing than the currently-used perspex insulators. The power supply circuit was also redesigned to allow operation at a high repetition rate.

## Data Availability

Data underpinning this paper are available from the University of Strathclyde at 10.15129/bb63a923-54a5-40c7-9c76-6a95a894db76.
